# Anti-inflammatory effect of resveratrol attenuates the severity of diabetic neuropathy by activating the Nrf2 pathway

**DOI:** 10.18632/aging.202830

**Published:** 2021-03-26

**Authors:** Wanli Zhang, Huan Yu, Qingxia Lin, Xiaoqian Liu, Yifan Cheng, Binbin Deng

**Affiliations:** 1 Department of Pediatrics, Tianjin Children's Hospital, Beichen, Tianjin, P.R. China; 2 Department of Neurology, First Affiliated Hospital of Wenzhou Medical University, Wenzhou, P.R. China; 3 Department of Psychiatry, First Affiliated Hospital of Wenzhou Medical University, Wenzhou, P.R. China

**Keywords:** anti-inflammatory, resveratrol, diabetic neuropathy, Nrf2 pathway

## Abstract

The mechanisms underlying the development of neuropathy associated with diabetes mellitus are not fully understood. Resveratrol, as a nonflavonoid polyphenol, plays a variety of beneficial roles in the treatment of chronic diseases such as Alzheimer's disease, coronary heart disease and obesity. In our study, the role of nuclear erythroid 2-related factor 2 (Nrf2) in resveratrol-mediated protection against streptozotocin-induced diabetic peripheral neuropathy (DPN) was investigated, and the antioxidant effect of resveratrol in diabetic peripheral nerves was studied. The STZ-treated model mice were divided into two groups. The resveratrol group was intragastrically administered 10 ml/kg 10% resveratrol once a day until the 12th week after STZ injection. The vehicle-treated mice were injected with the same volume of DMSO. Analysis of the effects of resveratrol in DPN revealed the following novel findings: (i) the pain and temperature sensitivities of diabetic mice were improved after treatment with resveratrol; (ii) Nrf2 expression was increased in the diabetic peripheral nerves of resveratrol-treated mice, and NF-KB pathway inhibition protected nerves upon resveratrol treatment in peripheral neuropathy; and (iii) resveratrol modulated the anti-inflammatory microenvironment of peripheral nerves by increasing Nrf2 activation and the expression of p-p65, and these changes may have been responsible for the neuroprotective effect of resveratrol in DPN, which was confirmed by Nrf2 knockout in diabetic mice. Overall, this study demonstrates that resveratrol may attenuate the severity of DPN by protecting peripheral nerves from apoptosis by inhibiting the NF-KB pathway and increasing Nrf2 expression.

## INTRODUCTION

Diabetic peripheral neuropathy (DPN), a complication of diabetes mellitus (DM), is the most common clinical peripheral neuropathy, with a prevalence of 50–60% [1, 2]. The main clinical symptoms are symmetrical pain and sensory abnormalities in the distal extremities. DPN occurs in various types of diabetes, and the condition worsens gradually with age [3]. DPN seriously affects the quality of life of patients with diabetes. However, keeping blood sugar levels stable is the only recommendation for preventing and alleviating symptoms [4]. It is extremely important to further understand the pathogenesis of DPN and find an effective drug.

The pathogenesis of DPN is not fully understood. In the course of DM, the accumulation of peroxides and the activation of the polyol pathway promote cellular metabolic disorders. In the peripheral nerves, DPN is characterized by Schwann cell metabolic dysfunction and a lack of neurotrophic support. Persistent abnormal metabolism and cAMP levels leads to Schwann cell apoptosis in the peripheral nerves [5, 6]. Long-term high glucose levels in the body lead to excessive production of highly active molecules, such as active nitrogen and reactive oxygen species, in the peripheral nerves and oxidation to a degree that exceeds the scavenging ability of the body, resulting in a series of oxidative stress reactions [7].

Resveratrol is a polyphenol found in natural plants and fruits such as Veratrum, Polygonum cuspidatum, grapes and peanuts. Resveratrol has a variety of beneficial effects, such as preventing oxidative stress injury, regulating blood sugar levels, inhibiting inflammation, and improving insulin resistance, in the treatment of many chronic diseases, such as Alzheimer's disease, DM, coronary heart disease and obesity [8]. Resveratrol binds to lipoproteins, enhances the antioxidant activity of these proteins, and reduces the oxidation of low-density lipoprotein and the deposition of cholesterol in arteries, thus reducing the risk of hyperlipidemia and atherosclerosis [9–11]. Resveratrol activates Sirt1 and NAD+-dependent deacetylase, improves mitochondrial function, activates endogenous antioxidant stress nuclear factor 2 related factor 2 (NF-E2-related factor2, Nrf2), increases the expression of phase ii detoxification enzymes, scavenges free radicals and alleviates tissue oxidative damage [12, 13].

As a classic signaling pathway, the NF-KB signaling pathway is widely involved in the expression and regulation of genes related to cell survival, proliferation and differentiation and plays an important role in inflammation, cell proliferation, oxidative stress, and apoptosis. The activation of the NF-KB pathway is closely related to inflammation, autoimmune diseases, tumors and other diseases [14, 15]. Previous studies on pancreatic cancer cells have found that blocking the interaction between keap1 and Nrf2 results in Nrf2 accumulation in the nucleus, a change in activation of the NF-KB pathway and resistance to malignant cell proliferation [16]. In a model of subarachnoid hemorrhage, NF-KB pathway activation is inhibited, the expression levels of TNF-α and IL-1β are decreased and the protein expression of keap1, Nrf2, and HO-1, which play a protective role in neurons, is increased [6, 17].

Resveratrol has low toxicity and widespread effects, so it has become a popular focus of many research fields at home and abroad. Recent studies have shown that resveratrol has antioxidative and immunoregulatory effects. In DM, long-term high glucose levels in the body lead to oxidative stress in the peripheral nerves [7]. In this study, the role of resveratrol in DPN was further explored, and the antioxidant effect of resveratrol in diabetic peripheral nerves was studied. Inflammation plays an important role in the development of diabetes. In our mouse model of DPN, the relationship between Nrf2 and NF-KB was further investigated by regulating the activation of the Nrf2 pathway.

## RESULTS

**Figure 1:**
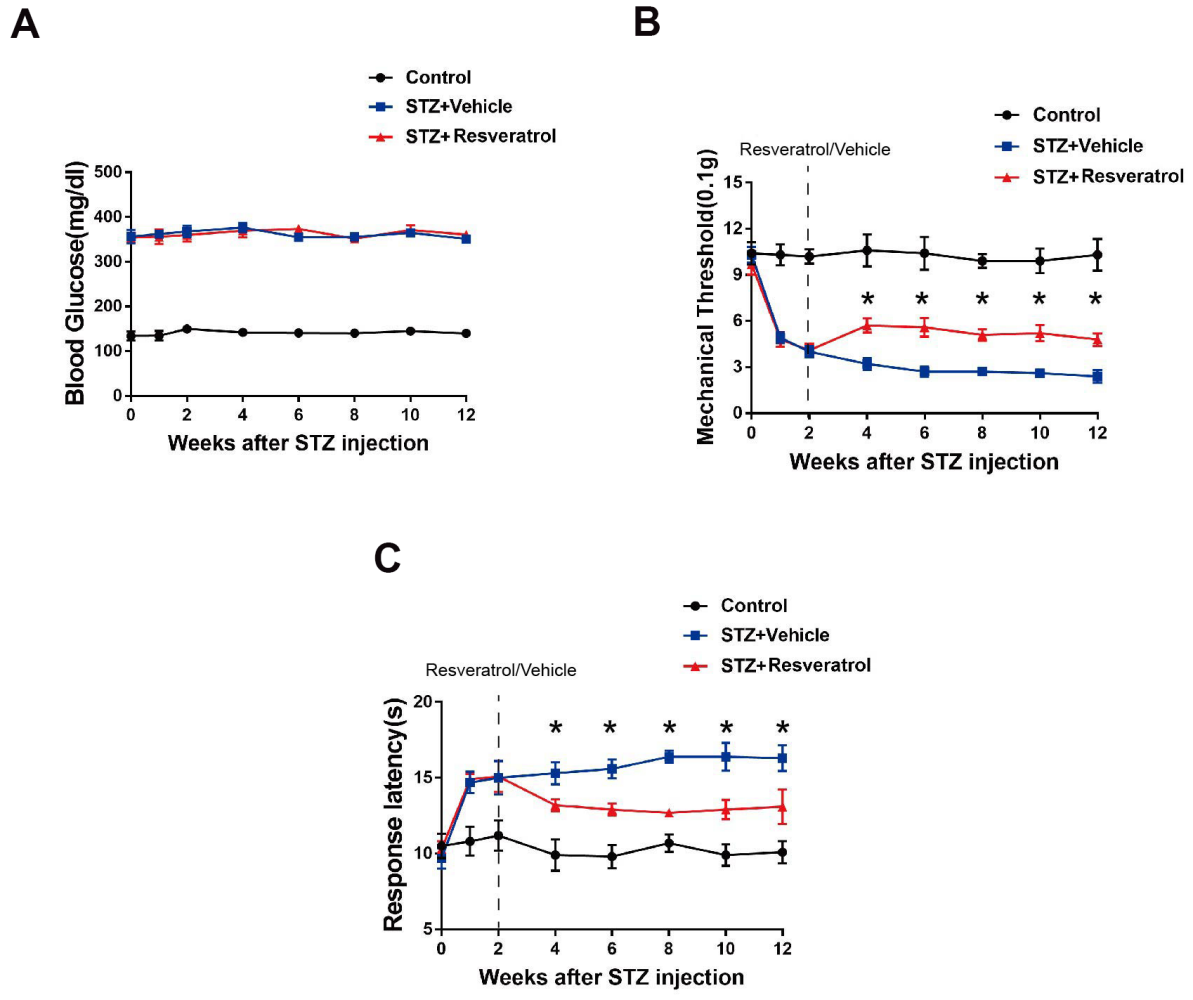
(**A**) Effect of fisetin on blood glucose in STZ+Vehicle and STZ+Resveratrol mice. (**B**) Mechanical nociceptive thresholds: ordinates represent the filament weight (0.1g) in which the animal responds in of presentations. (**C**) Thermal nociceptive threshold: the axis of ordinates represents the time (seconds) the animal takes to withdraw its paw. (Control group represents mice without administered with streptozotocin. 2 weeks after induction, mice were treated with Resveratrol or Vehicle (DMSO), n = 10 mice for each group, data are represented as the mean ± SEM.)

At the beginning of the study, a mouse model of diabetes was established, and the mice in which blood sugar levels were induced to a defined level by intraperitoneal STZ injection were identified as diabetic mice. The diabetic mice were randomly divided into the resveratrol treatment group and vehicle (DMSO) treatment group, and the corresponding drugs were administered during the second week after STZ injection. The blood glucose levels of each group of mice were observed, and the study found that the blood glucose levels of diabetic mice, including those treated with resveratrol and vehicle, were higher (Figure 1A) than those of control mice (mice that did not receive STZ injection). In our experiment, the two groups of mice were further observed after resveratrol or vehicle administration. The mechanical threshold values of resveratrol-treated mice were higher than those of vehicle-treated diabetic mice (Figure 1B). At the same time, the response latencies of resveratrol-treated mice were decreased, which showed that the thermal sensitivity of diabetic mice was increased after resveratrol intervention (Figure 1C). Abnormalities in pain and temperature sensitivity were observed in diabetic mice over the course of disease, while the pain and temperature sensitivity of diabetic mice were improved after resveratrol intervention.

**Figure 2:**
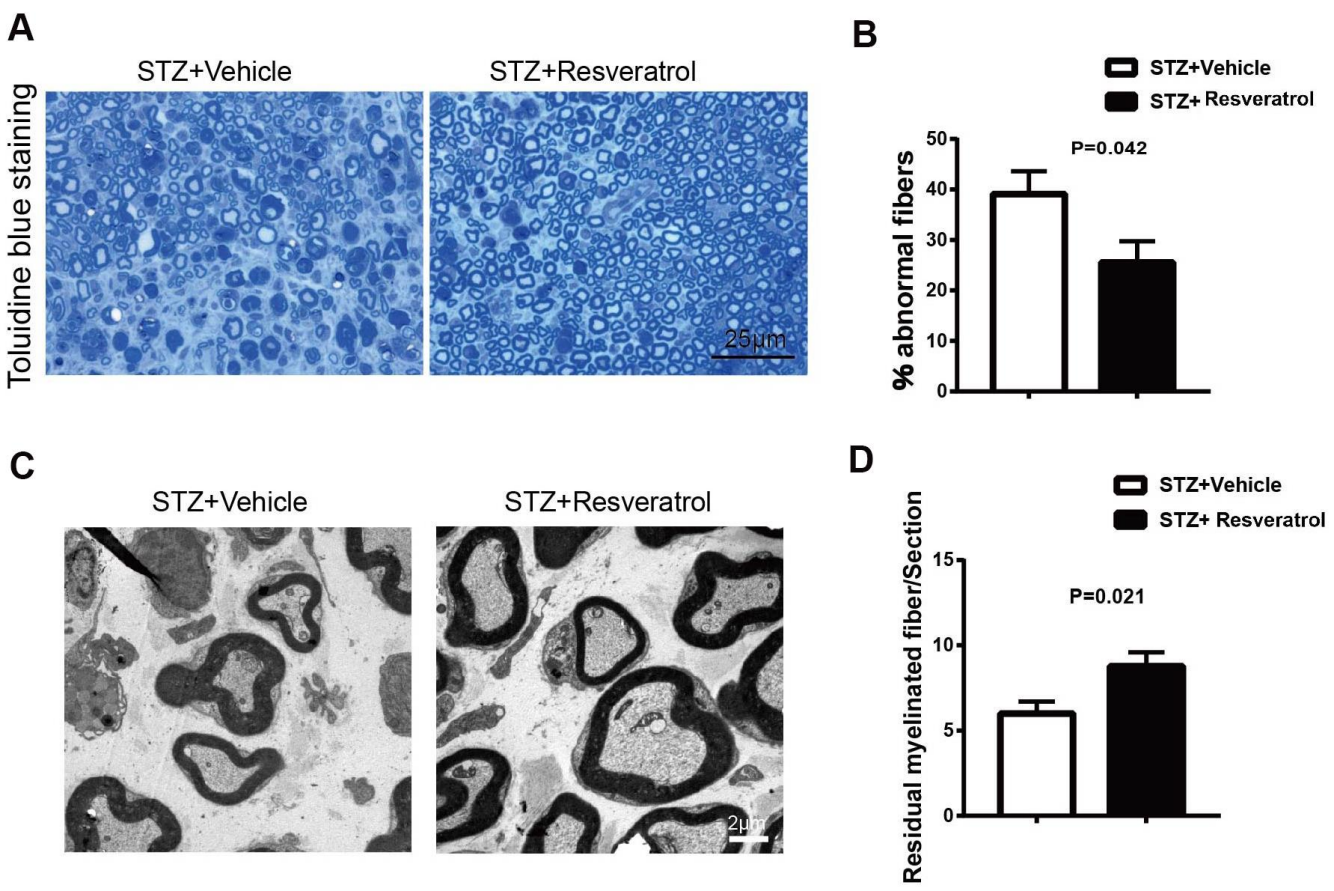
(**A**, **B**) Toluidine blue staining of semi-thin transverse sections of STZ+Vehicle and STZ+Resveratrol mice at 12 weeks after STZ injection. The percentage of abnormal fibers was quantitated (n = 3 mice per group; data are presented as means ± SEM). (**C**, **D**) Morphological assessment of axonal and myelin in sciatic nerves from STZ+Vehicle and STZ+Resveratrol mice. Representative electron micrographs from transverse ultra-thin sections of the sciatic nerves from STZ+Vehicle and STZ+Resveratrol at 12 weeks after STZ injection. (n = 3 nerves for each group, data are represented as the mean ± SEM).

Diabetic mice were sacrificed 12 weeks after STZ intervention, and the peripheral nerves of resveratrol- and vehicle-treated were further observed. The results of toluidine blue staining showed that compared to those of resveratrol-treated mice, the peripheral nerves of vehicle-treated mice were severely damaged, showing high degrees of myelin disintegration and axonal degeneration (Figure 2A, 2B, p=0.042). The same changes were also found by TEM. The number of myelinated fibers remaining in the peripheral nerves of vehicle-treated mice was decreased (Figure 2C, 2D, p=0.021).

**Figure 3:**
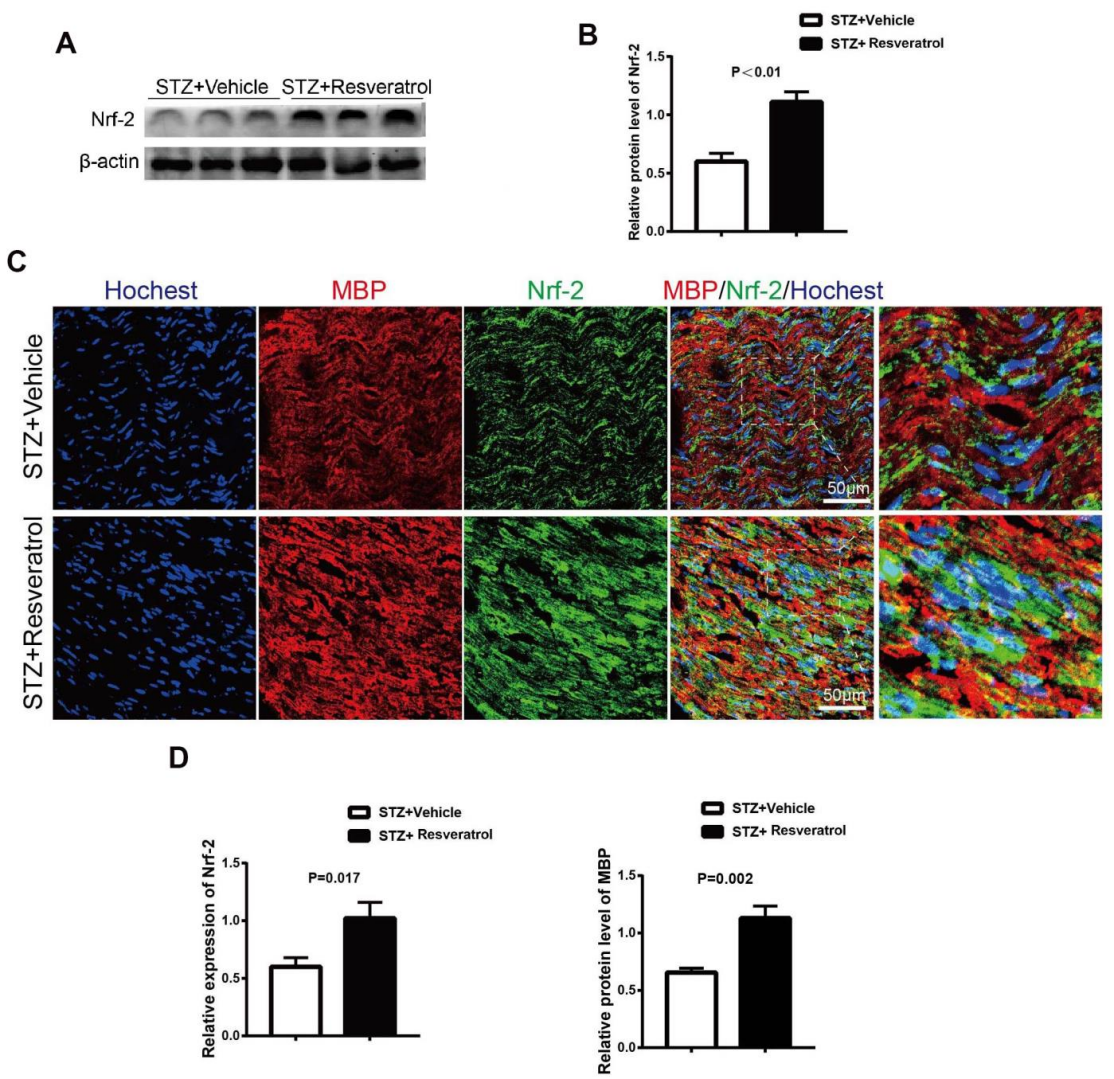
(**A**, **B**) Quantification by Western blot analysis showed that the expression of Nrf2 increased in STZ+Resveratrol mice at 12 weeks after STZ injection, compared with the never from STZ+Vehicle mice (n = 3 nerves for each group, data are represented as the mean ± SEM). (**C**, **D**) Immunofluorescence in longitudinal sections of STZ+Vehicle and STZ+Resveratrol sciatic nerves for Nrf2 (green) at 12 weeks after STZ injection. Nrf2 and MBP was increased in STZ+Resveratrol. (n = 3 nerves for each group, data are represented as the mean ± SEM).

Recent studies have shown that resveratrol has antioxidant effects. In DM, long-term high glucose levels in the body lead to oxidative stress in the peripheral nerves [7]. As an important *in vivo* endogenous antioxidant *in vivo*, Nrf2 was further explored in this study. The protein level of Nrf2 was detected in the peripheral nerves of resveratrol- and vehicle-treated mice. The results showed that the protein expression level of Nrf2 in the peripheral nerves of resveratrol-treated mice was increased (Figure 3A, 3B, p<0.01). In the peripheral nerves, Nrf2 fluorescence was observed, and the staining results suggested that the Nrf2 level in mice was increased after resveratrol administration compared to that in vehicle-treated mice; this was consistent with the results of protein electrophoresis (Figure 3C, 3D, p=0.017). Mbp is a myelin structural protein that is crucial for myelination [18–20]. Our study found that the expression of MBP was increased in mice treated with STZ-resveratrol, which suggested that resveratrol has a protective effect on myelin (Figure 3D, p=0.002).

**Figure 4:**
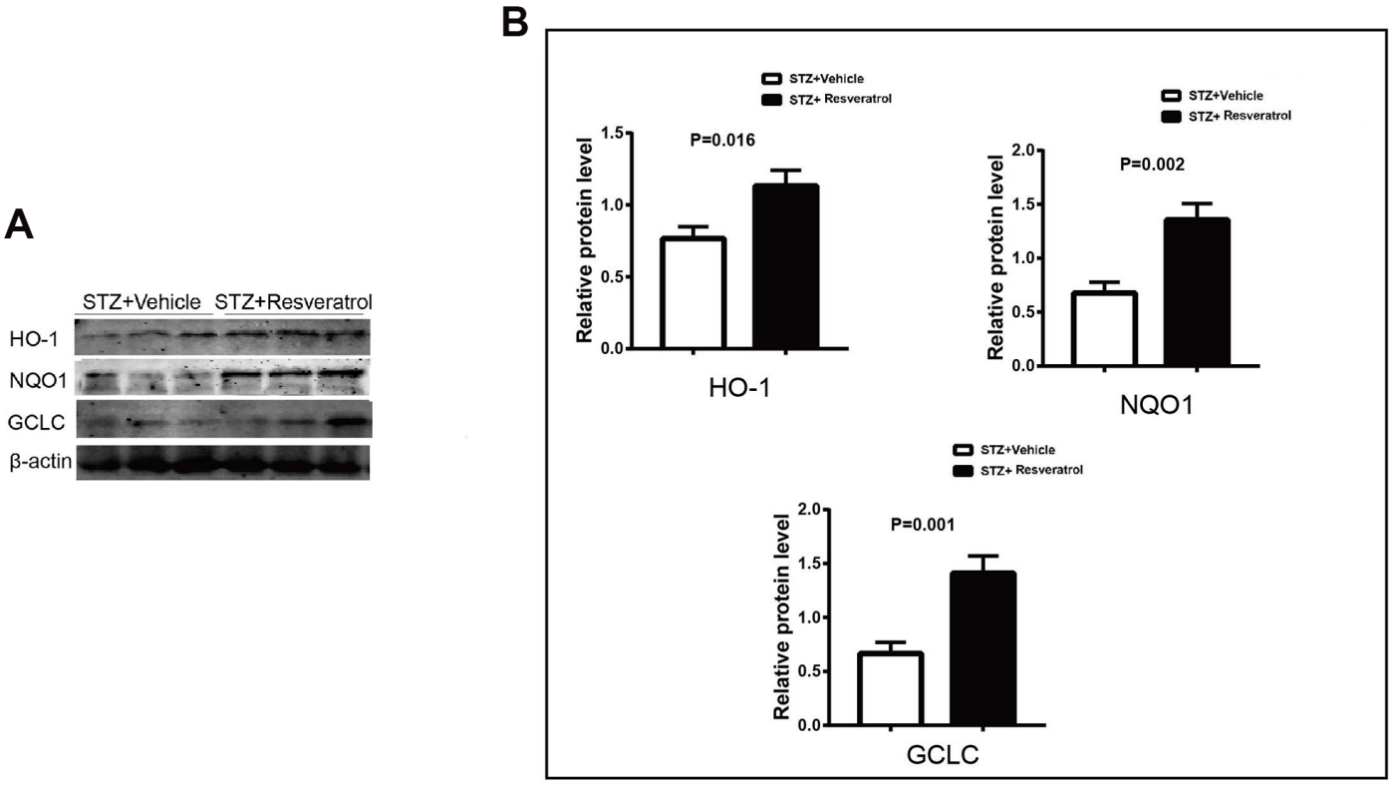
(**A**, **B**) Western blot analyses of lysates of HO-1, NQO1, GCLC from STZ+Vehicle and STZ+Resveratrol mice using the indicated antibodies at 12 weeks after STZ injection (n = 3 nerves for each group, data are represented as the mean ± SEM).

The antioxidant effect of Nrf2 is achieved through the activation of many antioxidant enzymes. Nrf2 can upregulate the expression of the antioxidant enzymes heme oxygenase-1 (heme oxygenase-1) and quinone oxidoreductase-1 (NAD (P) H:quinone oxidoreductase l, NQ01). The related antioxidant enzyme indexes were further detected, and the results showed that resveratrol interfered with increases in HO-1, NQO1 and GCLC in the peripheral nerves of mice (Figure 4A, 4B, p=0.016 for HO-1, p=0.002 for NQO1 and p=0.001 for GCLC).

**Figure 5:**
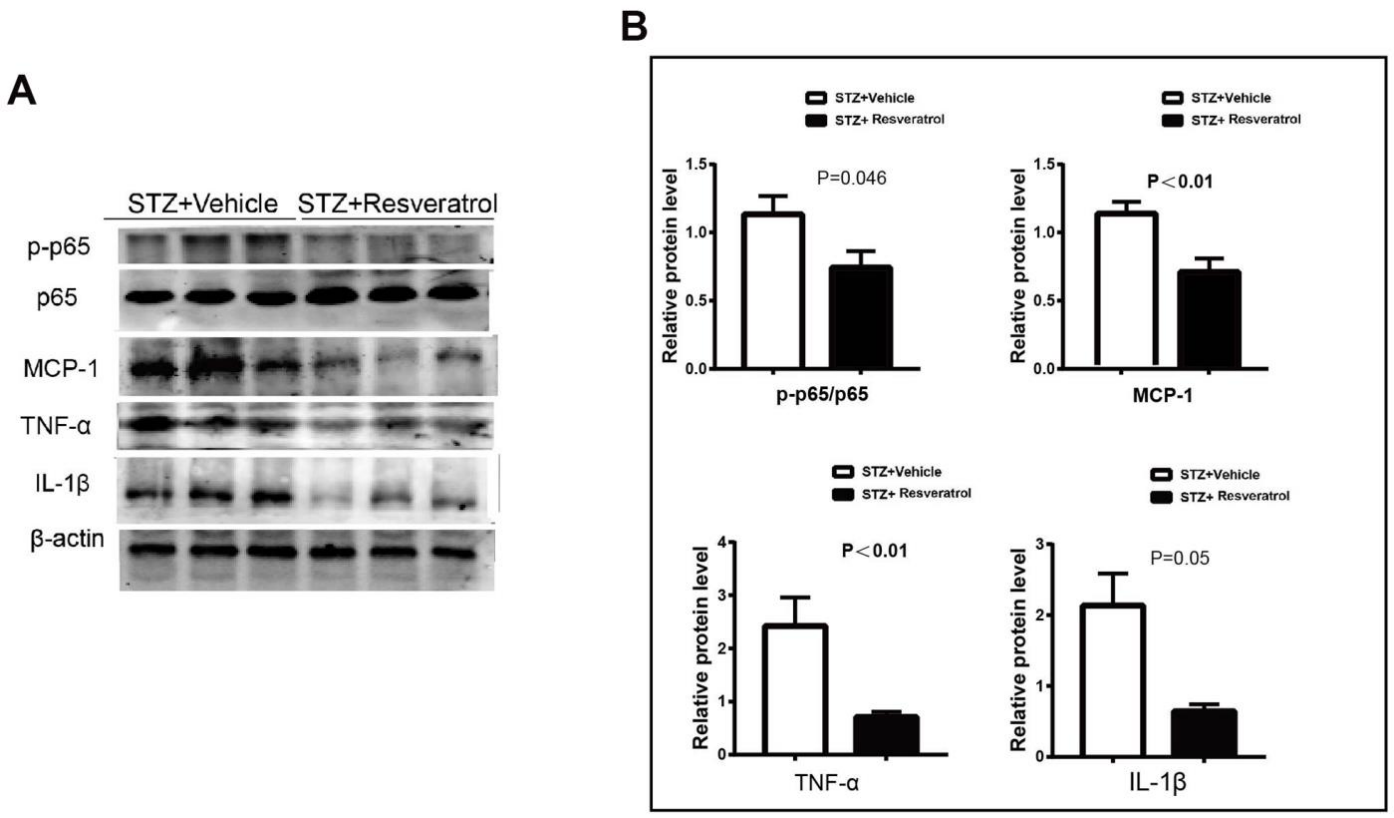
(**A**, **B**) Western blot analysis of lysates of p-p65/p65, MCP-1, TNF-a, IL-1β from STZ+Vehicle and STZ+Resveratrol mice, Quantification by Western blot analysis showed that the activation of AKT and the Bax, Cleaved caspase3 decreased in STZ+Resveratrol mice at 12 weeks after STZ injection (n = 3 nerves for each group, data are represented as the mean ± SEM.).

Previous studies have shown that LPS induces the expression of genes related to oxidative stress in RAW cells. upregulates Nrf-2 and HO-1 expression, inhibits NF-KB (p65) phosphorylation and affects the secretion of TNF-α and IL-1 β [15, 17]. In our study, the protein expression of Nrf2 and related antioxidant enzymes was increased in resveratrol-treated mice with peripheral neuropathy, and NF-KB and related inflammatory factors were further investigated. It The protein levels of p-p65/p65 were increased (p=0.046), and the levels of MCP-1, TNF-α, and IL-1 β were also found to be upregulated in the peripheral nerves of vehicle-treated mice (Figure 5A, 5B, p<0.01 for MCP-1 p<0.01 for TNF- α and p=0.05 for IL-1β).

**Figure 6:**
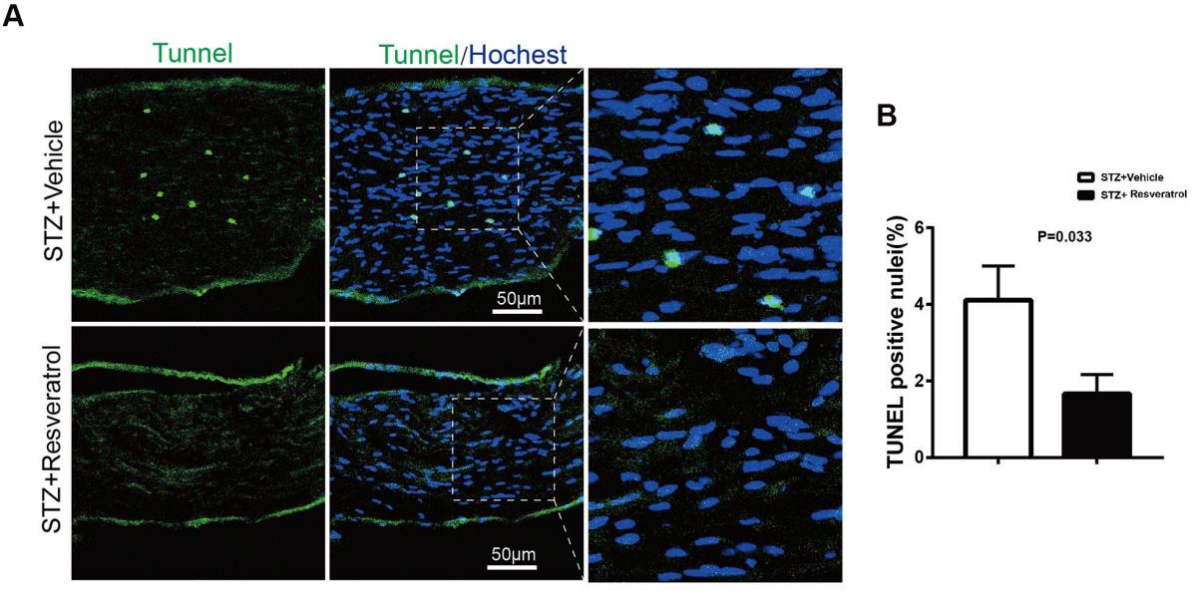
(**A**, **B**) TUNEL analysis (green) in longitudinal sections of STZ+Vehicle and STZ+Resveratrol mice stained with the nuclear dye Hoechst (blue) at 12 weeks after STZ injection. (n = 3 animals for each group, data are represented as the mean ± SEM). TUNEL, terminal deoxynucleotidyl transferase dUTP nick end labeling.

The level of apoptosis in the peripheral nerves is changed during DM. As the most important endogenous antioxidant *in vivo*, Nrf2 can reduce the production of ROS and apoptosis mediated by oxidative stress [21–23]. Apoptosis in the peripheral nerves of the two groups of mice was further explored. TUNEL staining showed that TUNEL-positive nuclear staining decreased after resveratrol intervention (Figure 6A, 6B, p=0.033). Additionally, the levels of Nrf2, as an important indicator of the oxidative stress pathway, were found to increase as the antioxidant levels in the peripheral nerves increased. To further explore whether the improvement in the peripheral nerves of the mice was dependent on the Nrf2 pathway, Nrf2 knockout mice were used.

**Figure 7:**
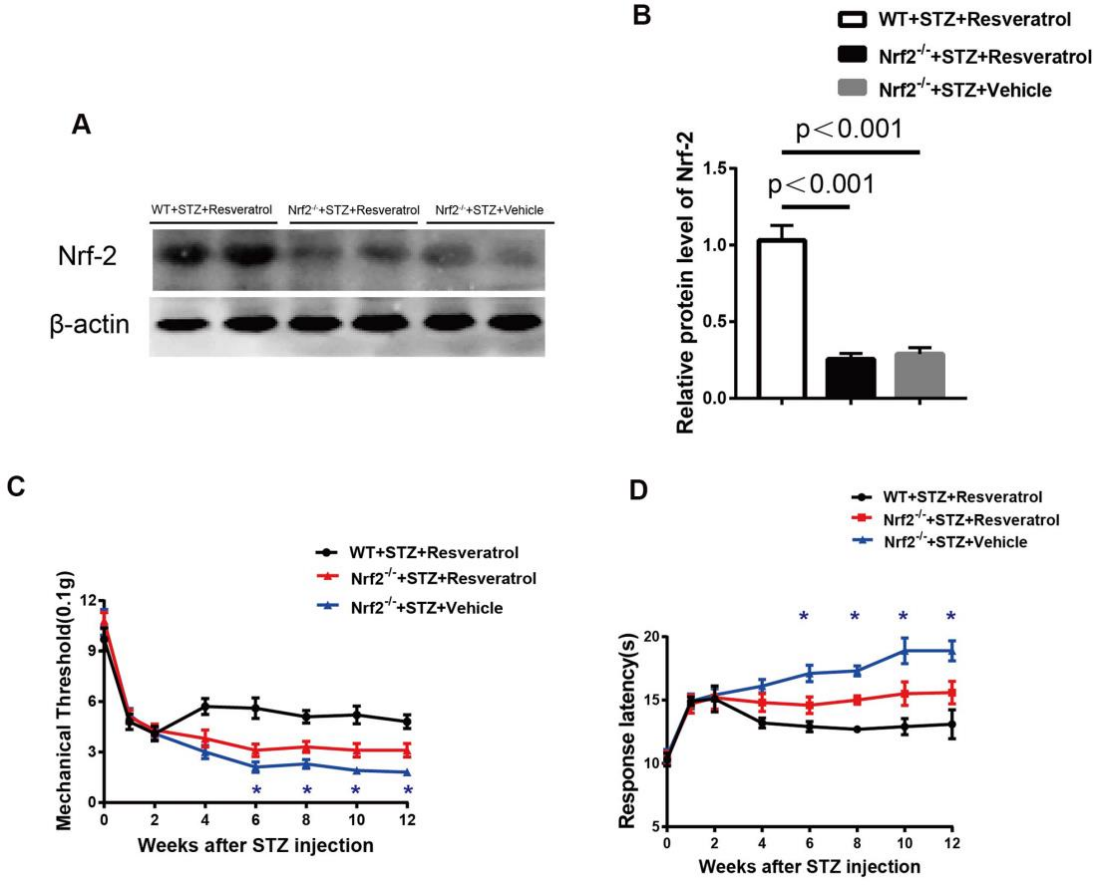
(**A**, **B**) Quantification by Western blot analysis showed that Nrf2 decreased in the Nrf2-/-+STZ+Vehicle and Nrf2-/-+STZ+Resveratrol nerve, compared with WT+STZ+Resveratrol mice at 12 weeks after STZ injection (n = 3 nerves for each group, each nerve was repeated 3 times, data are represented as the mean ± SEM). (**C**, **D**) Mechanical nociceptive thresholds: ordinates represent the filament weight (0.1g) in which the animal responds in of presentations. Thermal nociceptive threshold: the axis of ordinates represents the time (seconds) the animal takes to withdraw its paw. (2 weeks after STZ induction, mice were treated with Resveratrol or Vehicle (DMSO), n = 10 mice for each group, data are represented as the mean ± SEM).

The protein level of Nrf2 was detected, and the results showed that it was increased in the peripheral nerves of wild-type (WT) mice compared with those of of Nrf2 knockout mice treated with resveratrol and the peripheral nerves of Nrf2 knockout mice treated with vehicle (Figure 7A, 7B, p<0.01).

The study revealed that after resveratrol or vehicle intervention, compared with those of the peripheral nerves of Nrf2 knockout mice treated with resveratrol, the mechanical threshold values of diabetic Nrf2 knockout mice treated with vehicle were decreased (Figure 7C) when pain was evaluated and that the response latencies of diabetic Nrf2 knockout mice treated with vehicle were shorter (Figure 7D) when thermal sensitivity was tested.

**Figure 8:**
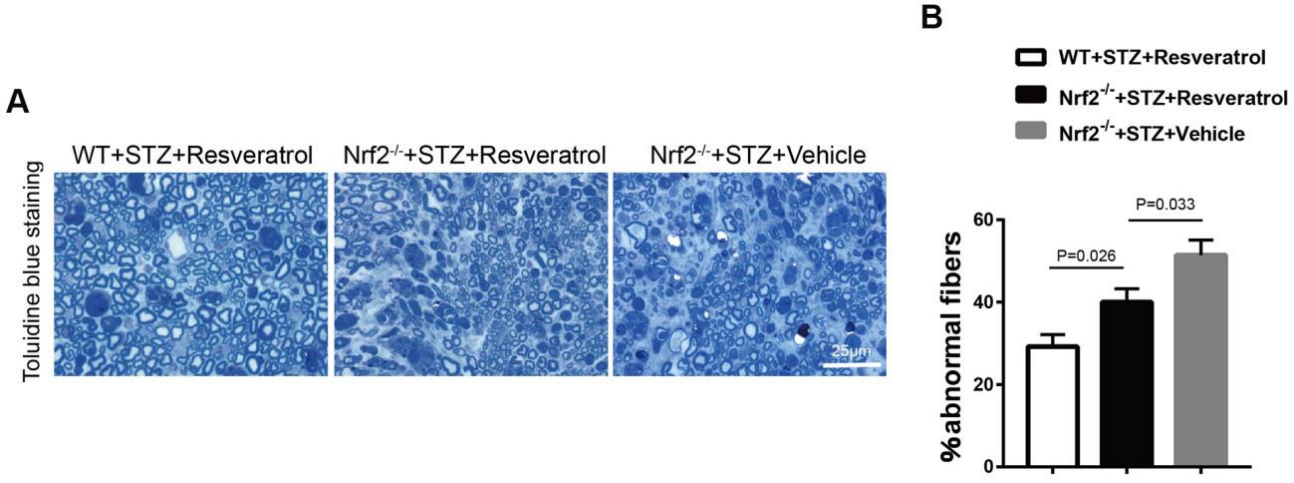
(**A**, **B**) Toluidine blue staining of semi-thin transverse sections of Nrf2-/-+STZ+Resveratrol, Nrf2-/-+STZ+Vehicle and WT+STZ+Resveratrol mice at 12 weeks after STZ injection, The percentage of abnormal fibers was quantitated, and the difference between the three groups was significant (n = 3 mice per group; data are presented as means ± SEM).

**Figure 9:**
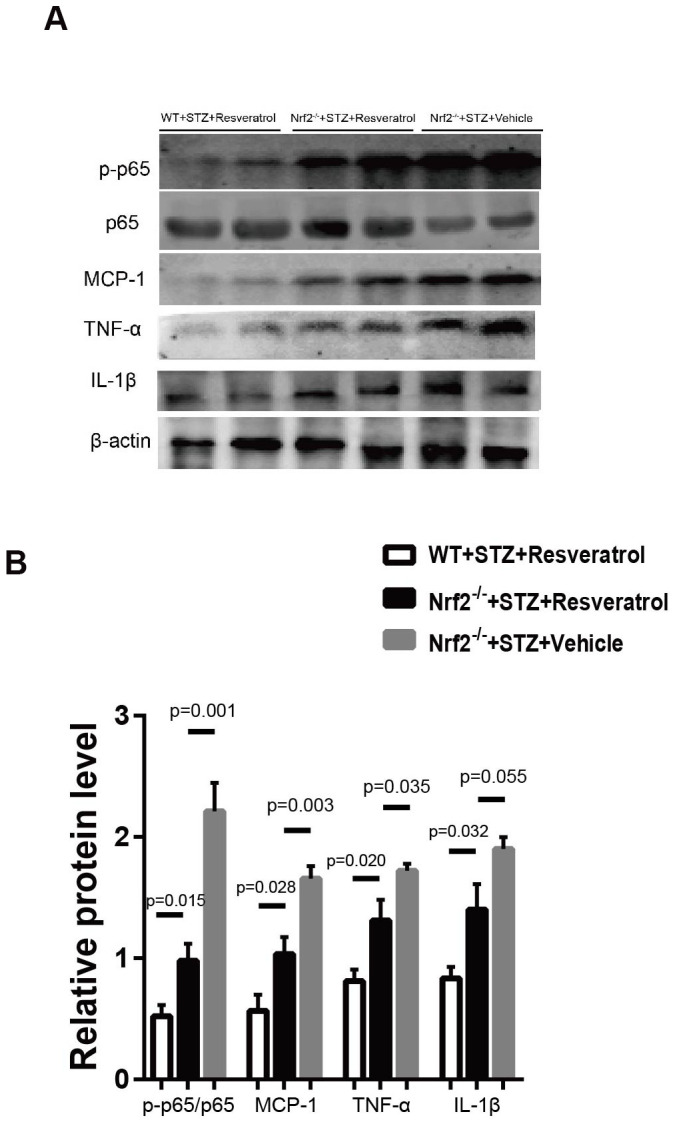
(**A**, **B**) Western blot analyses of lysates of p-p65/p65, MCP-1, TNF-a, IL-1β; from Nrf2-/-+STZ+Resveratrol, Nrf2-/-+STZ+Vehicle and WT+STZ+Resveratrol mice using the indicated antibodies at 12 weeks after STZ injection(n = 3 mice per group; data are presented as means ± SEM).

Toluidine blue staining was performed, and it was found that the peripheral nerves of Nrf2 knockout mice treated with vehicle were more severely damaged than those of Nrf2 knockout mice treated with resveratrol (Figure 8A, 8B, p=0.033). To confirm the effect of resveratrol on peripheral nerve inflammation in Nrf2 knockout mice, the levels of inflammation-related factors were measured. The results showed that the expression of MCP-1, TNF-α, and IL-1β was decreased (Figure 9A, 9B, p=0.003 for MCP-1, p=0.035 for TNF-α and p=0.055 for IL-1β) in resveratrol-treated Nrf2 knockout mice compared with vehicle-treated Nrf2 knockout mice. The protein level of p-p65/p65 was also detected in this experiment. In diabetic mice, resveratrol intervention affected the activation of NF-KB, which may be related to the inflammatory microenvironment of diabetic peripheral nerves (Figure 9A, 9B, p=0.001).

## DISCUSSION

In this study, the role of resveratrol in DPN was explored. The Nrf2 pathway was activated in the peripheral nerves, the levels of related antioxidant enzymes were increased, altering the level of oxidative stress in diabetic peripheral nerves, and Schwann cells apoptosis was regulated, inhibiting the destruction of diabetic peripheral nerves.

Resveratrol is a nonflavonoid polyphenol that was first extracted from the roots of *Veratrum grandiflorum* by Takaoka in 1940 and was also extracted from the roots of *Polygonum cuspidatum* by Nonomura in 1963 [24]. In 1997, resveratrol was reported to have an inhibitory effect on multiple carcinogenic processes as a chemical prophylaxis [25]. The biological activities of resveratrol mainly include antiviral [26], neuroprotective [10], antioxidant [11] and anti-inflammatory activities [9]. The role of resveratrol in diabetic peripheral nerves was explored in our experiment. Our study found that resveratrol plays an important role in alleviating damage to the peripheral nerves of diabetic mice. In addition, the mechanism by which resveratrol affects diabetic peripheral nerves was studied, and the experimental results suggested that resveratrol may exert its important effects on diabetic peripheral nerves by activating the Nrf2 pathway. Resveratrol can inhibit the growth of cells by inducing cell cycle arrest, including by downregulating BCL-2 and Bcl-xL expression, to activate cysteine-containing aspartic acid proteases. In this study, the apoptosis level in diabetic peripheral nerves induced by resveratrol was explored. Resveratrol effectively reduced the level of apoptosis in the peripheral nerves by activating Nrf2.

Oxidative stress is the imbalance between oxidative processes and antioxidative defense in the body; persistent hyperglycemia in the body leads to excessive production of reactive oxygen free radicals and active nitrogen free radicals, leading to oxide scavenging disorders and ultimately tissue damage [27]. Oxidative stress can induce insulin resistance, which can cause insulin resistance by activating a variety of redox-sensitive kinases. Excess ROS directly impair the function of islet cells and promote islet B cell apoptosis [23]. When the endogenous antioxidant stress factor Nrf2 is activated, the expression of phase II detoxification enzymes and the level of oxidative stress increase [12, 13]. Our study suggests that resveratrol has a protective effect on diabetic peripheral nerves by activating the Nrf2 pathway and related oxidases. Resveratrol plays an important role in peripheral nerves through Nrf2. Nrf2 knockout mice were further used to confirm the important role of Nrf2 under the action of resveratrol.

The Nrf2 and NF-KB pathways are important pathways that regulate the intracellular redox response and inflammatory balance. The interactions between these pathways involve a series of complex molecular interactions. Nrf2 is a key factor in intracellular oxidative stress. The NF-KB signaling pathway is widely involved in the expression and regulation of related genes, such as cell survival, proliferation and differentiation, and plays an important role in inflammation, cell proliferation, oxidative stress, apoptosis and so on [12, 17, 28]. Nrf2 deletion may lead to the enhancement of NF-KB activity and promote the production of cytokines, while NF-KB can regulate the transcription and activity of Nrf2. When the keap1 gene is knocked out by siRNA in human umbilical vein endothelial cells, the expression of Nrf2-mediated antioxidant genes is upregulated, and TNF-mediated NF-KB activity and the NF-KB signaling pathway are inhibited. Keap1 knockout induces the expression of Nrf2-dependent phase II enzymes and then inhibits TNF-induced ROS accumulation in cells. ROS are important factors necessary for NF-KB activation [29–31].

In our experiment, we found that inflammation-related factors were secreted in the peripheral nerves of diabetic mice. Resveratrol decreased the expression of related factors, including MCP-1, TNF-α, and IL-1 β, in the peripheral nerves, and NF-KB was also found to be inhibited in the diabetic peripheral nerves of resveratrol-treated mice. In this study, the Nrf2 gene was knocked out, which altered inflammatory microenvironment in the peripheral nerves; this change may have influenced the protective effect of resveratrol on the peripheral nerves.

The results of our study suggest that resveratrol modulates NF-KB by increasing Nrf2 activation and the expression of antioxidant enzymes, which might be responsible for the neuroprotective effect of resveratrol in DPN.

## MATERIALS AND METHODS

### Mice

Nrf2^-/-^ and Nrf2^+/+^ CD1/ICR mice were obtained from Johns Hopkins University. CD1/ICR mice were purchased from the Beijing Vital River Laboratory. Nrf2^-/-^ and Nrf2^+/+^ mice were genotyped by PCR. The primers are listed in Table 1. The mice used for the experiments were bred under controlled conditions, i.e., a 12-h light/dark cycle, 60 ± 10% relative humidity, and 22 ±1° C. Animal Experimental Ethical Inspection of Laboratory Animal Centre, Wenzhou Medical University (WYDW2019-0499).

**Table 1:** Primer.

**Primer**	**Forward**	**Reverse**
Nrf2	TGGACGGGACTATTGAAGGCTG	GCGGATTGACCGTAATGGGATAGG

### Antibodies and chemicals

The following antibodies and reagents were used: Nrf2 antibody (Abcam, Cambridge, UK, ab31163), NQO1 antibody (Abcam, Cambridge, UK, ab2346), GCLC antibody (Abcam, Group, Wuhan, China, 14241-1-AP), P-65 antibody (Cell Signaling Technology, MA, USA), p-P65 antibody (Cell Signaling Technology, MA, USA), MCP-1 antibody (Abcam, ab8101), TNF-α antibody (Proteintech 60291-1-IG), β-actin antibody (Proteintech, 60008-1), Hoechst stain (1:100, Invitrogen), TUNEL apoptosis assay kit (Beyotime Institute of Biotechnology, C1089), secondary antibodies for Western blotting (Rockland Immunochemicals, PA, USA), and fluorescein-isothiocyanate-conjugated secondary antibodies (Jackson ImmunoResearch, PA, USA).

### Modeling and drugs

Before model establishment, fasting for 12 h and body weight measurements, some mice were randomly selected for the normal control group. The mice that were not selected for the normal control group were injected once into the left lower abdominal cavity with 120 mg/kg body weight 1% streptozotocin (STZ), and the mice in the normal control group were injected with the same amount of citrate buffer solution. Blood glucose levels in samples taken from the tails of the mice were measured 72 h after STZ injection, and two random blood glucose measurements greater than 300 mg/dl indicated that the model was successfully established.

The successful model mice (two random blood glucose measurements greater than 300 mg/dl) were randomly divided into two groups and treated during the second week after STZ injection. The resveratrol group was intragastrically administered 10 ml/kg 10% resveratrol once a day until the 12th week after STZ injection. The vehicle-treated mice were intragastrically administered the same volume of DMSO once a day until the 12th week after STZ injection.

### Behavior tests

Von Frey filaments (Stoelting, IL, USA) were used to measure mechanical threshold values. Each mouse was placed on a customized platform. Von Frey filaments were applied to the center area of the plantar surface of the hindpaw in ascending order until an obvious reflex was induced. The filaments were each applied for 2 s, and the interval between applications was 15 s. The paw withdrawal threshold was defined as the force of the filament that caused at least three reactions of the hind paw over five trials.

As previously described [18], thermal withdrawal thresholds were detected using the plantar test (Hargreaves Apparatus, Ugo Basile Biological Instruments, Gemonio, Italy). A light source was placed under a glass floor, and the light source was directed at the center of the hind paw of each mouse. When the mouse retracted its paw, the latency to withdrawal was recorded. Each mouse underwent three stimulations with an interval of at least 15 min. The average threshold of the three tests was recorded as the thermal withdrawal threshold (in seconds).

### Western blot analysis

Nerves were harvested and transferred to tubes. Proteins were extracted using a protein extraction kit (Applygen Technologies Inc., China, P1250). Proteins were separated by SDS-PAGE and then transferred to PVDF membranes. The membranes were incubated with primary antibody for 12 h at 4° C followed by secondary antibodies for 1 h at 37° C and then scanned with an Odyssey Infrared Imaging System (LI-COR, NE, USA).

### TUNEL staining

TUNEL staining was performed using a one-step TUNEL apoptosis assay kit. Longitudinal sections of the nerve were incubated with the reaction mixture for 2 h at 37° C after permeabilization with 0.1% Triton X-100 (Olympus FV1000).

### Immunofluorescence staining

Nerves were dissected and cut into 8-μm-thick longitudinal sections. After the sciatic nerve sections were blocked with 0.3% Triton X-100 PBS, they were incubated with primary antibodies for 12 h and then with secondary antibodies for 2 h. The sections were observed under a fluorescence confocal microscope (Olympus FV1000).

### Toluidine blue staining and electron microscopy

As previously described [19], the mice were sacrificed, and the sciatic nerve was perfused with 4% glutaraldehyde. After the sciatic nerve was stained with cesium tetroxide and dehydrated in gradient ethanol solution, it was embedded in resin. The sciatic nerve was cut into semithin sections (0.5 μm), stained with toluidine blue and observed under a microscope (Olympus, Tokyo, Japan).

The sciatic nerve was cut into ultrathin sections (50-nm-thick) for electron microscopy analysis. The ultrathin sections were stained with lead citrate and uranyl acetate and then observed by transmission electron microscopy (TEM, JEM-1230).

### Statistical analysis

Each experiment was performed using at least three mice. The values are expressed as the means ± standard errors of the means (SEMs), and the data were analyzed using SPSS 24.0 software. Data from different groups were analyzed using Student’s t test, ANOVA, or the Mann-Whitney U-test, as appropriate. Differences were considered significant when P < 0.05.
